# Supplementary material for: Biphasic Fermentation of Trapa bispinosa Shells by Ganoderma sinense and Characterization of Its Polysaccharides and Alcoholic Extract and Analysis of Their Bioactivity
Source: Molecules. 2024 Mar 11;29(6):1238. doi: 10.3390/molecules29061238 (PMC10975738; doi:10.3390/molecules29061238)
Supplement: Supplementary file 1 [file molecules-29-01238-s001.zip › molecules-2815090-supplementary.pdf]

Table S1: Fermentation conditions

Level	X_1：Time of Fermentation（d）	X_2：Temperature of Fermentation（℃）	X_3：Humidity of Fermentation（%）
-1	1.5	12.5	70
0	2	15	75
1	2.5	17.5	80

Table S2 Primer sequences of genes related to NF-κB pathway

Gene name	Forward Primer（5'→3'）	Reverse Primer（5'→3'）
p50	AAATGGGAAACCGTATGAGCCTGTG	GTTGTAGCCTCGTGTCTTCTGTCAG
p52	GCTGATGGCACAGGACGAGAAC	CAGGTGGTTGGTGAGGTTGATGAC
p65	TCTGCCG AGTAAACCGGAAC	CAGGCTAGG GTCAGCGTATG
p100	GATCCGCTCCATGCTAATGTGAATT	AGCTTTTCCAAAAAAGCTCCATGC
ikk α	GTACAGCGACAGCACAGAGATGG	TGACCAAACAGCTCCTTGAGAACAC

Table S3 Primer sequences of genes related to Nrf2/Keap1-ARE pathway

Gene name	Forward Primer（5’→3’）	Reverse Primer（5’→3’）
keap1	CGGGGTACCATGGGCCAGCCA	CCCAAGCTTTTAAATCTTATCGT
gpx2	CGGGGTACCATGGGCATGGACTT	ATTTGCGGCCGCGTTTTTCTTAAC
gpx5	GCAATCCTGTCCTCACCCTT	ACGCCATCAGGTCCCACT
gclc	GTCCTCAGGTGACATTCCAAGC	TGTTCTTCAGGGGCTCCAGTC
gclm	AAGCTGGTCATCAACGGGAAAC	GAAGACGCCAGTAGACTCCACG

Table S4 Analysis of variance of regression model

Source	df	Sum of Squares	Mean Square	F-value	p-value
Model	9	4.71	0.5234	186.62	< 0.0001
A-Time	1	0.3668	0.3668	130.78	< 0.0001
B-Temperature	1	0.2336	0.2336	83.28	< 0.0001
C-Humidity	1	0.1897	0.1897	67.65	< 0.0001
AB	1	0.0027	0.0027	0.9456	0.3632
AC	1	0.0028	0.0028	1	0.3503
BC	1	0.0376	0.0376	13.42	0.008
A^2	1	2.89	2.89	1029.16	< 0.0001
B^2	1	0.3195	0.3195	113.92	< 0.0001
C^2	1	0.3859	0.3859	137.58	< 0.0001
Residual	7	0.0196	0.0028		
Lack of Fit	3	0.0117	0.0039	1.95	0.264
Pure Error	4	0.008	0.002		
Cor Total	16	4.73			

Table S5 FT-IR analysis results

Wavenumber（cm^{-1}）		Assignment
P1	P2	
3421.55	3423.76	O-H stretching vibrations
2926.97	2926.69	CH_2 Asymmetric stretching vibration
	1640.94	Disordered amide I
1628.53		β-lamellar amide I
	1457.52	CH_2 in polysaccharide structure
	1399.56	CH_2 rocking vibration
1372.41	1365.7	C-H rocking vibration
1155.04	1153.93	C-O-C asymmetrical stretching vibration
1081.56	1081.29	Stretching vibration of β-glucose
1024.62	1024.92	Contraction vibration of α-glycosyl bond
840.1	840.11	α-glycoside bond
	773.61	Ring breathing vibration
703.91	703.27	OH Non-plane bending vibration
609.3		skeletal vibration
577.79	580.71	skeletal vibration
444.52	445.21	skeletal vibration

Table S6 UPLC-MS/MS analysis results

Class	Compound Name	Content of Relative Percentage (%)		Trend
		AE1	AE2	
Polyphenols	Ellagic acid	1.3619	1.5374	+
	6-Gingerol	0.4508	0.2086	-
	Moracin C	0.0531	0.0035	-
	3-Galloylquinic acid	0.2907	0.2438	-
	Methyl gallate	0.0693	0.0879	+
	4'-Prenyloxyresveratrol	0.0646	0.0465	-
	Vanillin	0.9579	0.8217	-
	p-Octopamine	7.5469	2.6392	-
	1,3,6-Tri-O-galloyl-β-D-glucose	0.9584	0.9117	+
	4-Nitrocatechol	0.2325	0.1646	-
	Hamamelitannin	0	0.0392	+++
	beta-Glucogallin	3.7628	2.2445	-
	Ethyl 4-hydroxybenzoate	0.627	0.7222	+
Flavonoid	Pinocembrin	0	0.028	+++
	Chalconaringenin	1.7557	2.7809	+
	Naringenin	2.7557	0.9932	-
	Eriodictyol	0.0748	0.1155	+
	Cianidanol	0.3539	0.3104	-
	Gallocatechin	0.3822	1.5642	+
	Myricetin	0.5848	0.0227	-
	Dihydromyricetin	0.437	0.0987	-
	Astragalin	0.0488	0.1258	+
	Cynaroside	0.0824	1.1661	+
	Homoorientin	0.0418	0.1306	+
	Puerarin	0.0918	0.0386	-
	Maltol	0.5859	0.6003	+
	Kazinol A	0.0062	0.053	+
	Quercetagetin	0.0057	0.0727	+
	Kuwanon H	0.017	0	---
	Aromadendrin	0.1142	0.1173	+
	Epicatechin	0.0901	0.0655	-

Continued Table S6

Class	Compound Name	Contencontent of Relative Percentage(%)		Trend
		AE1	AE2	
Alkaloids	DL-Alanine	0.2884	0.2736	-
	Indole	2.1023	3.0304	+
	O-Acetylethanolamine	5.7329	6.2421	+
	Kynurenic acid	0.2477	0.2269	-
	Chimonanthine	0	0.0542	+++
	Solasodine	0.0475	0.0929	+
	D-Aspartic acid	0.1139	0.1156	+
	DL-Tyrosine	0.5731	0.4724	-
	3',4'-Anhydrovinblastine	0	0.0755	+++
	Crotonoside	12.6806	8.9563	-
	Piperlonguminine	0.6964	0.7833	+
	3-Methylindole	0.0752	0.0917	+
	Isoquinoline	0.0284	0.0755	+
Triterpenoids	3-Oxopomolic acid	0.0902	0.1196	+
	Ganoderic acid Jb	0.003	0.1034	+
	Ganoderic acid F	0.0052	0.1303	+
	Ganoderic acid H	0.0049	0.5265	+
	Ganoderol A	0.0027	0.829	+
	Ganoderal A	0.0112	0.0892	+
	Arjunolic acid	0.0195	0	-
	Betulinic acid	0	0.0158	+
	beta-boswellic acid	0	0.0174	+
	3-Epioleanolic acid	0	0.0943	+
	Diosbulbin D	0.0951	0.0721	-
	Miltirone	1.6128	2.0265	+
	Isorosmanol	0.103	0.0865	-
	Kaurenoic acid	6.0233	2.2	+
	Myrcene	0.0665	0	---
	2-(2-Hydroxy-2-propyl)-5-methyl-5-vinyltetrahydrofuran	0.1602	0.1116	-
	L(-)-Fenchone	6.3409	1.543	-
	Linderalactone	0.0468	3.0299	+
	trans-Caryophyllene	0.0829	0.0762	-
	Caryophyllene oxide	0.2554	0.2162	-
	Artemisinin	0.872	0.2241	-
	Lamiide	0	0.9038	+++
